# Sentiment Analysis Techniques Applied to Raw-Text Data from a Csq-8 Questionnaire about Mindfulness in Times of COVID-19 to Improve Strategy Generation

**DOI:** 10.3390/ijerph18126408

**Published:** 2021-06-13

**Authors:** Mario Jojoa Acosta, Gema Castillo-Sánchez, Begonya Garcia-Zapirain, Isabel de la Torre Díez, Manuel Franco-Martín

**Affiliations:** 1 Telecommunications Engineering, Engineering Faculty, University of Deusto, 48007 Bilbao, Spain; mbgarciazapi@deusto.es; 2 Department of Signal Theory, Communications, and Telematics Engineering, University of Valladolid, 47001 Valladolid, Spain; gemaanabel.castillo@alumnos.uva.es (G.C.-S.); isator@tel.uva.es (I.d.l.T.D.); 3 Department of Psychiatry, Río Hortega University Hospital, 47012 Valladolid, Spain; mfrancom@saludcastillayleon.es; 4 Department of Psychiatry, Zamora Healthcare Complex, 49022 Zamora, Spain

**Keywords:** mindfulness, stress, COVID-19, CSQ-8, natural language processing, deep learning, embedding, IMDB, swivel, neural networks

## Abstract

The use of artificial intelligence in health care has grown quickly. In this sense, we present our work related to the application of Natural Language Processing techniques, as a tool to analyze the sentiment perception of users who answered two questions from the CSQ-8 questionnaires with raw Spanish free-text. Their responses are related to mindfulness, which is a novel technique used to control stress and anxiety caused by different factors in daily life. As such, we proposed an online course where this method was applied in order to improve the quality of life of health care professionals in COVID 19 pandemic times. We also carried out an evaluation of the satisfaction level of the participants involved, with a view to establishing strategies to improve future experiences. To automatically perform this task, we used Natural Language Processing (NLP) models such as swivel embedding, neural networks, and transfer learning, so as to classify the inputs into the following three categories: negative, neutral, and positive. Due to the limited amount of data available—86 registers for the first and 68 for the second—transfer learning techniques were required. The length of the text had no limit from the user’s standpoint, and our approach attained a maximum accuracy of 93.02% and 90.53%, respectively, based on ground truth labeled by three experts. Finally, we proposed a complementary analysis, using computer graphic text representation based on word frequency, to help researchers identify relevant information about the opinions with an objective approach to sentiment. The main conclusion drawn from this work is that the application of NLP techniques in small amounts of data using transfer learning is able to obtain enough accuracy in sentiment analysis and text classification stages.

## 1. Introduction

Previous experienced gained from other countries in treating the pandemic caused by Covid-19, mainly China [1], has often shown that reactions resulting in stress and panic occur, both among health care professionals and patients affected, which makes it necessary to act quickly to improve their mental health [2,3]. It should be taken into account that, during the pandemic, health care professionals have often ended up completely exhausted as a result of the great pressure on them, including the increase in infection (at times with inappropriate protection) and contamination, as well as frustration, work overload, isolation, and the fact of dealing with patients and family members with negative, sometimes semi-aggressive sentiments, owing to the frustration they feel about being infected [2,4,5]. To this should be added external factors such as the fear of infecting their family members due to the work they do, which leads them to isolate themselves from the family in many cases [2]. Previous experience has even shown that the risk of post-traumatic stress disorder increases when professional quarantine is associated with the infection of a family member [6]. As a consequence, numerous mental health problems commonly emerge such as stress, anxiety, symptoms of depression, insomnia, distress, denial, and fears among health care professionals [4]. Indeed, these are considered to be a sector of the population that is particularly vulnerable to experiencing problems linked to mental health [7], not only during the pandemic but also afterwards, insofar as post-traumatic stress disorder often tends to persist [8].

On the other hand, we found in a recent study, that the incidence of anxiety disorders among staff working in the fight against Covid-19 was 23.04% (53/230), 2.17% of which were deemed to be serious, with such disorders seemingly being more common in women (25.67% vs. 11.63%) (Z = −2.008, *p* = 0.045]) [9]. It was also noted that the incidence and seriousness of anxiety disorders is higher among nursing staff than among doctors (26.88% vs. 14.29%, Z = −2.066, *p* = 0.039) [9].

All the aforementioned are of an importance that goes beyond mental suffering and loss of performance or health care worker satisfaction. Rather, it will also lead to a reduction in the capacity for immunological protection of their own bodies, which will expose them to greater risk of infection and fewer defenses against it, as we have known for many years now [10]. Some studies even show a high suicide rate among this group of health care professionals [11], who are being especially affected and at the forefront of the battle against COVID-19. This situation is fostering worldwide debate about the concerns felt by health care professionals regarding spreading and handling infection, exposure to their family members, health care colleagues, and work-related stress [12] at times of such uncertainty, with features common to each culture and country. This group of professionals deserves support services to help prevent suicide, which is why the initiative arose to offer mindfulness [13].

According to the studies [2,4,14], the authors said that it is necessary that the main hospitals supervise the health intervention programs (both psychological and psychiatric) from the initial phases of the pandemic, both preventive in nature and by way of intervention (even in times of crisis), in order to reduce problems affecting mental health. Moreover, it should serve to help improve psychological skills of medical professionals, with women who would seem to be the most affected, according to the different studies, being of special interest to nursing staff [9]. The fact that certain social sectors feel stigmatized owing to the high risk of becoming infected should be taken into account, in contrast to the superhuman effort required by those who are stigmatized individuals to survive [7,8]. In some cases, as occurred in the pandemic in Singapore, this perception of stigma affected 49% of health care workers [15].

In this study [16], the authors present that the strategic way of controlling the pandemic could be to integrate mental health into health care, especially if the latter is integrated. Within this context, an initiative is being considered based on the application of mindfulness [17] so as to be able to provide health care professionals from Castile and Leon with this, specifically those from the provinces of Valladolid and Zamora. This involves relaxation techniques to enable them to concentrate better to help deal with stress [18], anxiety [19], depression [19,20], or any other dysfunctional emotional states that may have affected them as a consequence of the situation arising from the Covid-19 pandemic [21,22]. Some interventions, such as Mindfulness-Based Cognitive Therapy (MBCT), have shown that they may protect individuals from depression [19] and may also facilitate a change in the objectives regarding self-regulation to help prevent suicide [20]. This work was complemented by the creation of a website www.massaludmental.es (accessed on 10 June 2021) [23], the aim of which was to provide details to help deal with stress experienced by professionals from SACYL (Castile and Leon Health care Service) [24], with a view to lending support to their mental and emotional wellbeing during the Covid-19 pandemic [25].

The authors of the works described above, proposed an online application that might enable this intervention to be accessed from the user’s own home, in order to help facilitate participation in these therapies. Therefore, during the first wave of the pandemic, health care professionals from Castile and Leon (Valladolid and Zamora) were provided with this therapy by way of support for helping to deal with stress [18], anxiety [19] or any other emotional or mental health disorders, thus contributing directly to suicide prevention [11,12,26]. The aim of this paper is to evaluate the degree of satisfaction and outcomes obtained from an initial approach to intervention through mindfulness applied online, in order to favor emotional control of professionals from the Castile and Leon Health Care System.

The quick growth of the performance of deep learning algorithms has made the Natural Language Processing a very important research topic to give solution to the health care concerns. Then, many works compiled in the systematic review of the state of the art in the application of sentiment analysis to address COVID-19 and other infectious diseases [27] show us the use of sentiment analysis techniques, mainly, lexicon-based models, machine learning-based models, and hybrid-based models, applied to disease mitigation with high interest to contribute in this area in the short term. In the same way, the use of transformer models has spread rapidly, since they can be adapted, using transfer learning techniques, to specific applications. In this sense, there are works such as the COVID transformer, which describe the use of clustering algorithms in conjunction with the coding provided for the specific model, to identify the trends in groups of tweets in the pandemic epoch [28].

In this way, sentiment analysis has been applied in different health-related contexts [29]. According to the source of information used, sentiment analysis can be divided into three groups: social networks [30], especially Twitter, ref. [31] refereed papers, and texts from interviews with family members [32]. As such, the study proposed in this paper fits into this last-mentioned type of interview/questionnaire. The purpose of the study was to apply artificial intelligence to texts gathered in a questionnaire about mindfulness therapy, with a view to defining new strategic lines of thought for such therapy in the future.

This paper comprises five sections. The Section 1 introduces the theme of mindfulness as a therapy in general, and also in times of pandemic in particular. Its aim is also to apply sentiment analysis to artificial intelligence in order to create strategies. The methods used to develop the work are described in the Section 2, mainly the swivel embedding word-encoding model, a classification model based on multi-layer perception and, lastly, the application of transfer learning based on IMDB data that can be freely downloaded. The Section 3 provides the results obtained, which are then discussed in the Section 4, with the conclusions being drawn in the Section 5.

## 2. Materials and Methods

Strategies involving information and communications technologies [25] were organized and implemented during the first lockdown in Spain from March to June 2020, so as to be able to lend support to health care workers in issues regarding mental health. In view of such uncertainty about the pandemic, a mindfulness course [12] was organized using the ZOOM videoconferencing platform [26] and disseminated via the website www.massaludmental.es (accessed on 10 June 2021) [21]. The aim of the course was to provide support for health care professionals from Castile and Leon in dealing with stress, anxiety, and other emotional disorders caused by having to face such an uncertain and unexpected situation as a pandemic, with a view to managing any thoughts and emotions that may lead to the contemplation of suicide or pursuit of an act of suicide. The intervention program got underway on 20 April and ended on 28 June 2020, and 24 sessions of 40 minutes’ duration were arranged twice a week, mornings and afternoons.

Online registration initially needed to be completed in order to send participants links to the course and session plans via email. At the time of registration, questions were asked as to what motivated them to pursue mindfulness, with registration itself being done online using GoogleForm and promoted via the website www.massaludmental.es (accessed on 10 June 2021) [21].

### 2.1. Survey

The survey based on (CSQ-8: Client Satisfaction Questionnaire) [27] is a customer satisfaction questionnaire made up of eight questions that are related to each other, in order to be able to measure the degree of satisfaction with treatment or intervention involving patients. Data such as age, gender, and place of work was also gathered with a view to describing the sample collected as appropriate. Additionally, participants were also requested to voluntarily provide answers to the following open questions (according to Table A2 of the Appendix A):

Q1: Share with us what you most liked about the attention received.
Q2: Share your suggestions and recommendations for improvement with us.

### 2.2. Study Participants and Procedure

The intervention program got underway on 20 April and ended on 28 June 2020, and 24 sessions of 40 minutes’ duration were arranged twice a week, mornings and afternoons.

Online registration initially needed to be completed in order to send participants links to the course and session plans via email. At the time of registration, questions were asked as to what motivated them to pursue mindfulness, with registration itself being done online using GoogleForm and promoted via the website www.massaludmental.es (accessed on 10 June 2021) [21].

Participants in the customers satisfaction questionnaire conducted for this study were health care workers or professionals from Castile and Leon who were actively employed during the first wave of the Covid-19 pandemic. Eighty-five percent of health care professionals were gainfully employed at the time, and of these, 93% were women, of whom a sample of 130 responded to open questions from the sentiment analysis carried out as follows: Q1 = 86 y Q2 = 68.

### 2.3. Labeling

The procedure used to label these questions was conducted as follows: Questions were sent to two professionals with experience in mental health, who then labeled each response as being either *positive, negative,* or *neutral* according to context and professional experience. A third professional from the area of technology then verified each labeled response and conducted a sentiment analysis using NPL.

#### Histograms of Labeled Text According to Sentiment Categories

Distribution of data according to class gives a clear idea of trends regarding sentiment in the categories taken into consideration for the questions selected for the case study. For this reason, data distribution graphs were put together for each of the questions asked in the questionnaire provided for the purpose of gathering such data.

The histograms corresponding to distribution of the number of responses per question and associated sentiment category are shown below.

**Figure 1:**
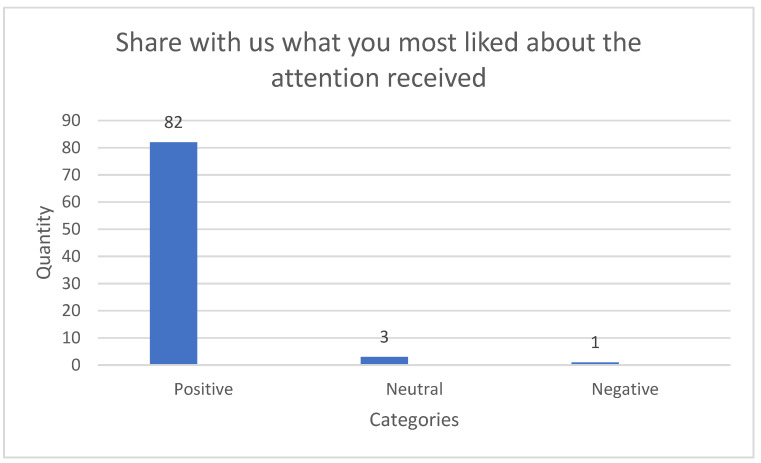
Histograms for the set of data corresponding to the question “Share with us what you most liked about the attention received”.

In the Figure 1, for the question “Share with us what you most liked about the attention received”, it is noted that most questions expressed a *positive* sentiment in comparison to the *negative* and *neutral* categories. The number of labels according to class for this question were: 82 positive, 3 neutral and 1 negative, out of 86 responses in total.

**Figure 2:**
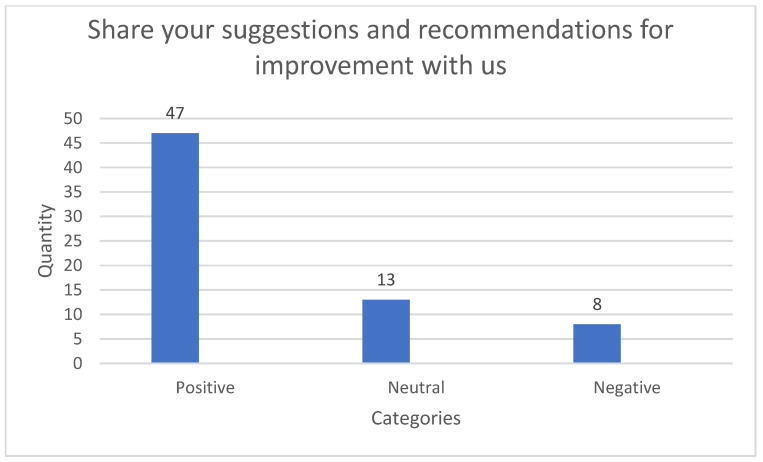
Histograms for the set of data corresponding to the question “Share your suggestions and recommendations for improvement with us”.

In the Figure 2, for the question “Share your suggestions and recommendations for improvement with us”, it is noted that most questions expressed a *positive* sentiment in comparison to the *negative* and *neutral* categories. The number of labels according to class for this question were: 47 positive, 13 neutral, and 8 negative ones, out of 68 responses in total.

### 2.4. NLP Techniques

**Figure 3:**
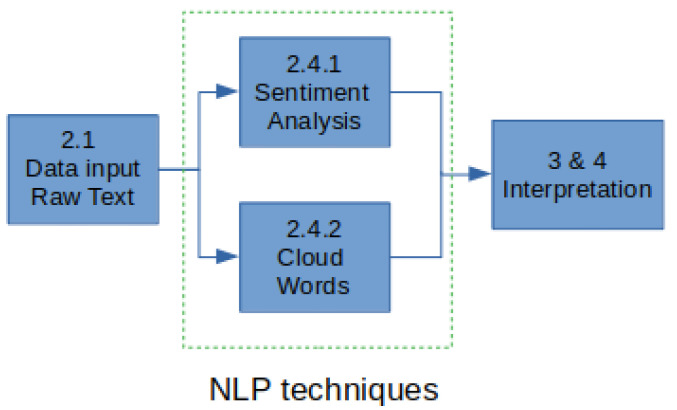
Block diagram of the Natural Language Processing model proposed.

Natural language processing techniques enable the content of a text to be analyzed in an automated way, with a view to extracting knowledge. Within the context of this research, we were mainly interested in sentiment analysis, as well as the computer graphic representation of the written text by users who have responded to the questionnaire. Therefore, two subsections are provided below describing the techniques used to achieve this objective in the Figure 3.

In the literature there are multiple models that allow the classification of texts, mainly in the application of Sentiment Analysis, for generic and specific applications. However, for the present application it was decided to use a pre-trained model, since the amount of data is very limited. Therefore, in the Submatrix-wise Vector Embedding Learner (Swivel) stage, we used the Swivel 20 pre-trained model, with google news database and a transfer learning on the multilayer perceptron classifier with the IMDB database. With this simple, but powerful structure, satisfactory results were obtained for the application under study.

#### 2.4.1. Sentiment Analysis

To analyze the sentiment in a text, it is initially necessary to represent it numerically, for which purpose the word embedding technique was used, as explained in Subsection Word Embedding. This technique encodes words in numeric vectors without losing information about the context, unlike the term frequency—inverse document frequency (TF-IDF) [33] in which the information of any word is based mainly on the frequency of appearance of it in the document.

It is important to highlight the fact that this technique requires a large amount of data in the training stage, which is why the decision was made to use transfer learning based on the IMDB data base [34], which is deemed suitable for these types of tasks and the details of which are explained in Subsection Classifiers. Once encoding in a numeric domain is available, a classifier is then trained that will be in charge of determining whether the text sentiment subject to study is positive, negative, or neutral. Details of the methods used for classification purposes are provided in Subsection Classifiers.

#### Word Embedding

Word embedding is a Natural Language Processing approach (NLP) [35] used to convert words into vector arrangements, with a view to capturing the semantic and syntactic relationship between words, thus simulating human learning of language vocabulary. The problem with encoding is one of great interest in this area of knowledge, which is why we should not just consider superficial forms of a text in order to represent words (e.g., using symbols, characters, word chains, sentences and documents), but also look for significant similarities (e.g., semantic or syntactic ones) between text fragments [36].

The main idea is to represent each word within a major body of text via a feature vector, in such a way as to be able to measure the similarity between vectors (e.g., words), using linear algebra (e.g., using cosine similarity [37]).

There are two categories of word embedding model: On the one hand, models on matrix factorization and, on the other, models based on sampling from a sliding window. This approach has proven to be very useful in tasks such as translation, analytics, and word similarity. There are currently several successful, well-known word embedding models such as GloVe [38] and Word2Vec [39], which have had a major impact on NLP research and inspired the construction of new word vectors based on stochastic gradient descent.

- Swivel Embedding

Swivel [40] (Submatrix-wise Vector Embedding Learner) is a model that proposes a hybrid between the shortcomings of the SkipGram Negative Sampling (SGNS) model [41] and GloVe [38]. On the one hand, it uses a co-occurrence matrix to calculate the PMI (point-wise mutual information) [42] between pairs of words for optimization purposes, thus reducing measurement using stochastic gradient descent between the weight vector point product (embeddings) for key words and words within context and PMI calculated by statistical counting of word co-occurrence within the corpus. One of the advantages is the chance to conduct distributed training, given that the nature of the proposal involves dividing the co-occurrence matrix and k matrices and thus extending the capacity for parallel training in a worker and central server configuration. This in turn enables *word embeddings* to be trained with a larger corpus, insofar as the computational cost is proportional to the size of the co-occurrence matrix, unlike in the case of the SGNS, where the cost is proportional to the size of the corpus. One of its advantages over GloVe is that it considers the weighting of non-observed co-occurrence, thus providing better vector representation for rare words.

#### Classifiers

To carry out the task of text classification in the three proposed categories: Negative, Neutral and Positive, we used multilayer perceptron with one hidden layer. This general structure was obtained using grid search method as hyperparameter adjustment. Finally, in both next cases, the IMDB data base was used to train and validate each classifier, the open response data base for test purposes, and swivel embedding to encode the text.

- MLP Multilayer Perceptron Structure 1

**Figure 4:**
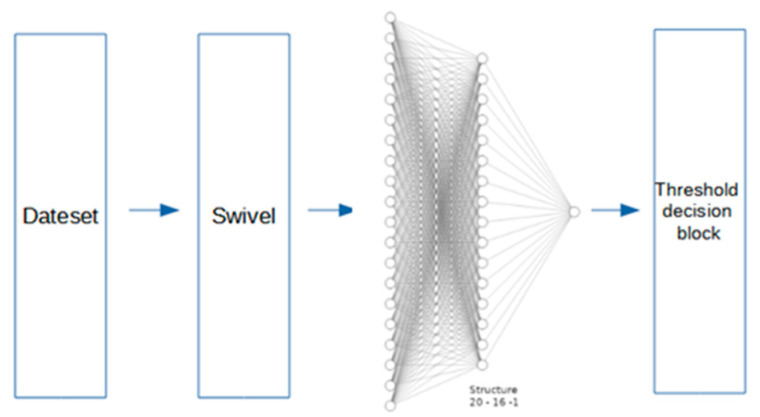
Outline of the proposal using MLP1.

This first proposed model is composed with a hidden layer of 16 neurons, each one with ReLU activation functions, and an output with one layer of a single neuron, activated by a sigmoid tangent function. An outline of the proposal is shown in Figure 4 below.

The decision was made obtaining a continuous value representing the degree of positive sentiment existing in a text fragment, i.e., a regressor between −1 and 1 is constructed from dichotomic features 0 and 1. This might customarily be used for a classification task.

- MLP Multilayer Perceptron Structure 2

**Figure 5:**
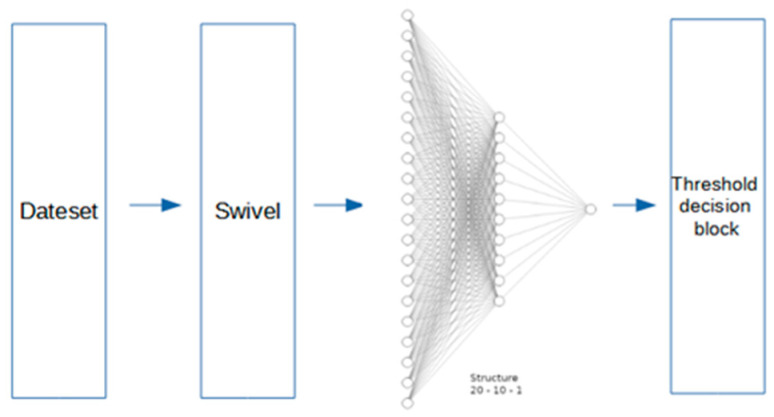
Outline of the proposal using MLP2.

The classifier now has a structure containing neurons for the input layer and 10 for the hidden layer. The activation function for the aforementioned layer is preserved in ReLU—a neuron in the output with sigmoid tangent function for the regression task. As in the case of the previous model, outputs correspond to a value between −1 and 1 in order to determine the degree of negativity or positivity of the text used in the input. Figure 5 shows the model proposed.

- Hyperparameters Adjustment

For the proposed models, the following hyperparameters can be identified as the main ones:

In Table 1, the activation function is fixed to the value RELU to reduce the computational cost of the hyperparameter grid search. On the other hand, the values that the hyperparameters can take are arranged based on previous experiences, such as the one shown in [43].

On the other hand, the parameters of the model, as the initialization of the weights, is done under a normal distribution of mean zero and variance one, as shown in the following probability density function Equation (1):

(1) $$f_{w}\left(w\right)=\frac{1}{\sqrt{\left(2\pi \right)}}e^{-\frac{{\left(w\right)}^{2}}{2}}$$

Finally, the two ones with the best performance were selected and to be compared in detail.

- Decision Model Based on Interval Comparison

**Figure 6:**
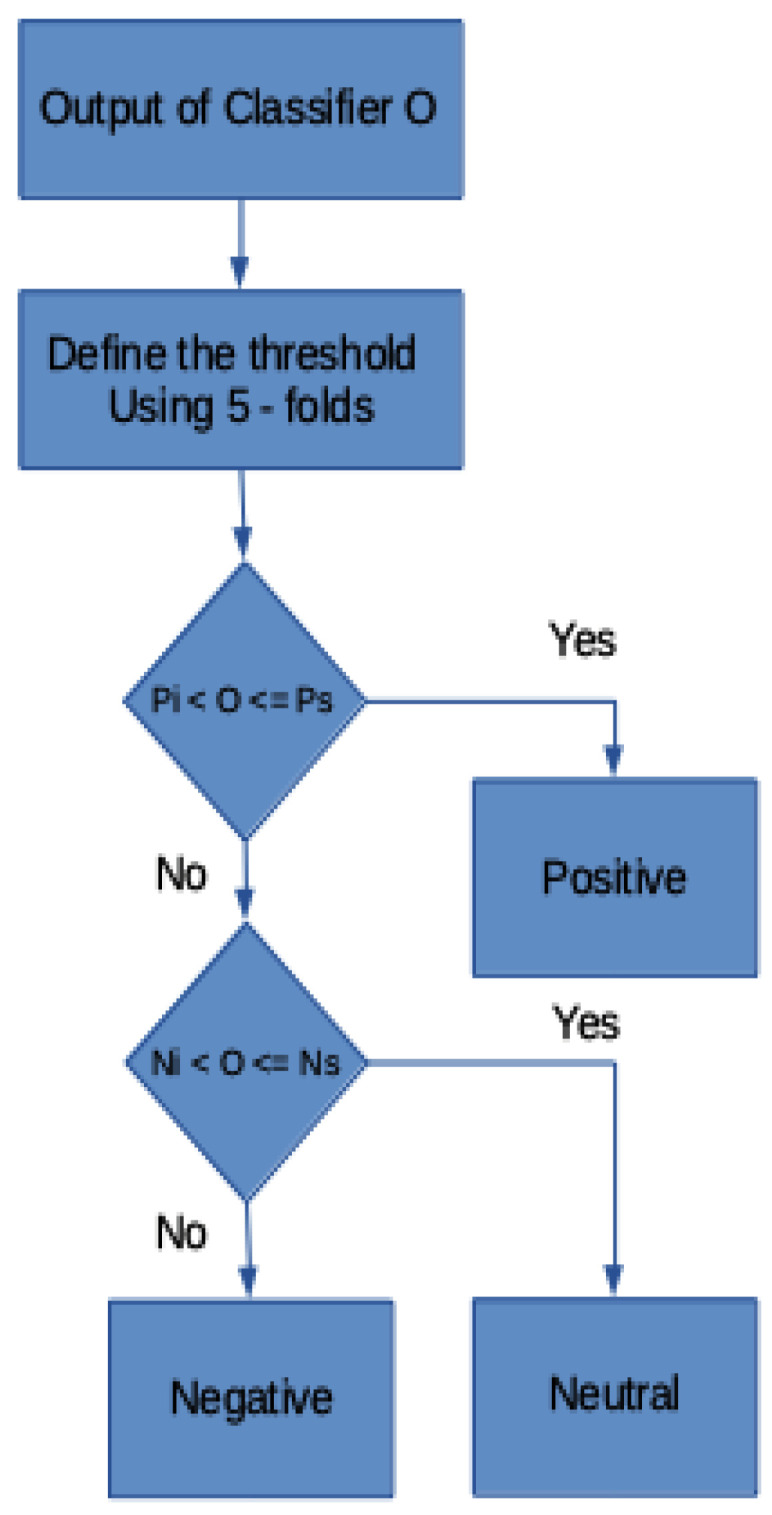
Flow chart of the decision block used to determine text sentiment.

Finally, a decision layer based on intervals is included in the output of the classification models proposed, the inferior neutral Ni and super neutral Ns limits of which were obtained via 5-fold cross validation, as the 0 values between the interval, −1, and 1 from the output of the model proposed need to be classified as either *positive, negative,* or *neutral*. It should be noted that the limits for the negative and positive categories were not explicitly established, as they were rejected with the two thresholds found. Moreover, attention should be drawn to the fact that the positive superior threshold is always 1 and the negative inferior threshold is always −1. The algorithm shown in the Figure 6 diagram is applied in order to achieve this objective.

Finally, in the Table 2 are presented the performance of the models proposed, measured based on the ground truth for test data—in this case, all the open responses from the instrument used to gather data.

#### 2.4.2. Word Clouds

A word cloud is a graphic representation of the relative frequency with which words appear in a text, i.e., the number of times a word is repeated within a text. It is very important to highlight the fact that words that are normally repeated in the language of the text being analyzed should be eliminated in order to obtain a significant representation. That is why determiners were eliminated from this application, as well as any special characters and punctuation marks. The threshold frequency that enables a word to appear within the computer graphics or configured word cloud is at least five repetitions. Finally, the image obtained will provide the word in a font size that is directly proportional to the number of times the word is repeated within a document or set of texts [44].

## 3. Results

In this section, we show the results obtained using the NLP text classifier, applied to determine the sentiment categories expressed by the persons who took the course and responded to the survey, the different categories being *negative sentiment, neutral sentiment,* and *positive sentiment*. We used the confusion matrix as a way to present the numbers, because they enabled us to understand the values in an intuitive way. Besides, the whole section is divided and analyzed into subsections in the same order as the methodology proposed.

### 3.1. NLP Techniques

Eighty-six and 68 registers were used for each case studied in order to obtain the results shown below, and these correspond to the responses to open questions from the instrument used to gather data from this research. To this end, four confusion matrices were obtained corresponding to the sentiment analysis models described in Subsection Classifiers together with an image from the computer graphics described in Section 2.4.2.

#### 3.1.1. Sentiment Analysis

Once the classification models proposed were trained and validated, the following results were obtained in the test stage—specifically, in the sentiment classification task for the input text. This corresponds to the opinions given by individuals who responded to the information in the survey gathered for the purpose of this research.

##### Decision Thresholds for Each Case

After the 5-fold cross validation was applied, the mean decision thresholds for each model proposed and the respective set of data were those obtained in Table 2 below, in addition to the mean accuracy obtained using the thresholds calculated [45].

As can be seen, the classifier based on MLP1 performs better in all cases in terms of accuracy in the classification of texts for the three categories *positive*, *neutral,* and *negative.*

##### Confusion Matrices for the Classifier Model Based on MLP1

With the thresholds obtained, the decision stage at the MLP model output was then run so as to thus determine the confusion matrix for Table 3 for the responses related to question 1.

Major errors from the positives classified as negatives classification model for responses to question 1: “SUPPORT”, it can be seen that the model tends towards error when inputs containing very little information are provided, which makes sense within the context of the question in the information-gathering instrument, although treated in isolation it does not, i.e., there is evidence of bias owing to the design of the information gathering. In other words, it does not take into account the context of the question in terms of decision-making—only the sentiment expressed by the user in the comment made.

With the thresholds obtained, the decision stage at the MLP1 model output was then run so as to thus determine the confusion matrix for Table 4 for the responses related to question 2.

Major errors from the positives classified as negatives classification model for responses to question 2: “Recorded sessions”. As in the case of texts corresponding to question 1, the same situation described previously is provided, in which it is stated that the NLP model does not take into account the context of the question in order for it to be objectively calculated at its output.

Major errors from the negatives classified as positives classification model for responses to question 2:

- I found the online connection to be very bad and the time was not very suitable for me
- Training mental health professionals so as to be able to amplify the effect
- Timetables sometimes did not fit in with my availability
- GREATER PRIVACY
- More timetable options

Additionally, and despite the fact that, broadly speaking, model performance proved to be successful, the bias evidenced by grammatical and spelling errors made by the person responding to the survey should be highlighted. As in the previous two cases, the incapacity of the model to take into account the context of the question being asked is also shown. This can be corrected in a relatively simple way, which focuses on correct design of the information-gathering instrument for analysis via natural language processing.

##### Confusion Matrices for the Classifier Model Based on MLP2

As in the case of the previous model, the decision stage at the MLP2 model output was then run so as to thus determine the confusion matrix for Table 5 for the responses related to question 1.

Finally, the decision stage at the MLP2 model output was then run so as to thus determine the confusion matrix for Table 6 for the responses related to question 2.

These two last-mentioned tables show the results obtained for the second model, selected via different iterations in the hyperparameter space and in particular the number of neurons in the hidden layer. It should be noted that performance in all cases was inferior to the best solution proposed.

##### Learning Curves for the Two Best Models Obtained

The graphs corresponding to the evolution of the *accuracy* metric are provided in order to observe the behavior of the best models obtained in the learning and validation stage, as the number of epochs increases in the course of running the algorithms.

**Figure 7:**
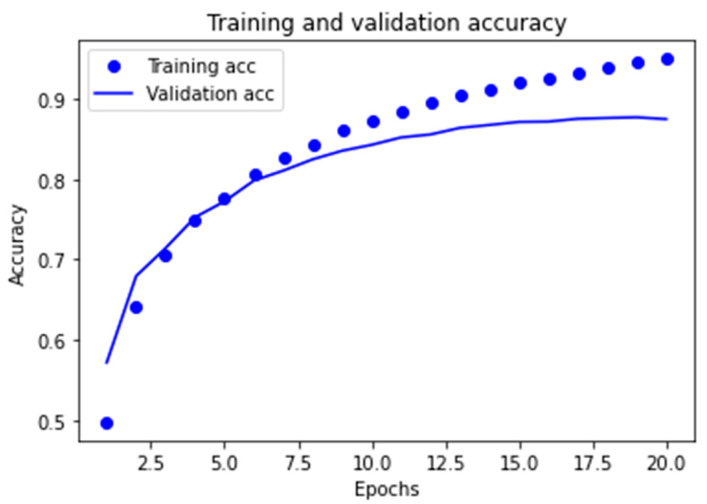
Learning graph for the MLP1 model with regard to the *accuracy* metric.

**Figure 8:**
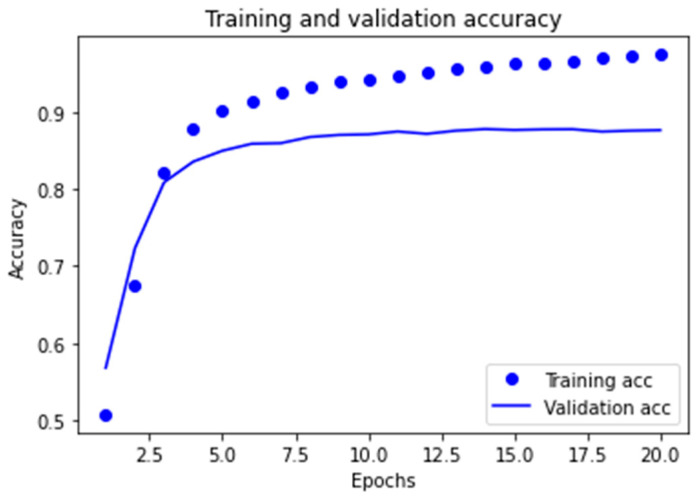
Learning graph for the MLP2 model with regard to the *accuracy* metric.

As can be observed in Figure 7 for the MLP1 model, the convergence of the algorithm is achieved in 10 epochs, in which the start of divergence of the learning curves and validation can be clearly seen. Furthermore, and despite the fact that the MLP2 model reaches convergence in a smaller number of epochs, mean performance is also less, as can be observed in Figure 8.

**Figure 9:**
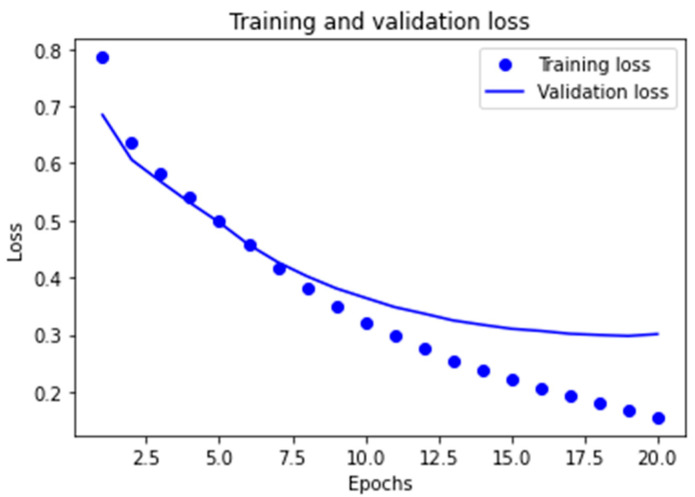
Flow chart of the decision block to determine text sentiment.

**Figure 10:**
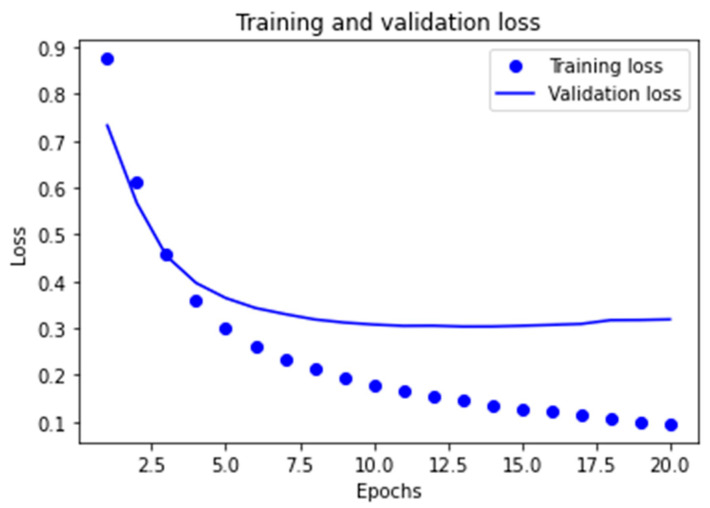
Flow chart of the decision block to determine text sentiment.

In the case of the cross-entropy loss used, behavior similar to the two cases subject to study was noted. In other words, Figure 9 and Figure 10 show us that the divergence for both models is initiated at the points corresponding to 10 and 5 epochs respectively, which entails overfitting for the models in terms of training data, thus limiting the capacity for generalization, which in turn is very important for this application.

Therefore, the early-stopping technique was applied for the two models in order to prevent their overfitting.

Thus, training of the MLP1 model was stopped in 10 training epochs, whereas the second MLP2 model was stopped in 5 epochs because the model training procedure had been implemented.

#### 3.1.2. Word Clouds

**Figure 11:**
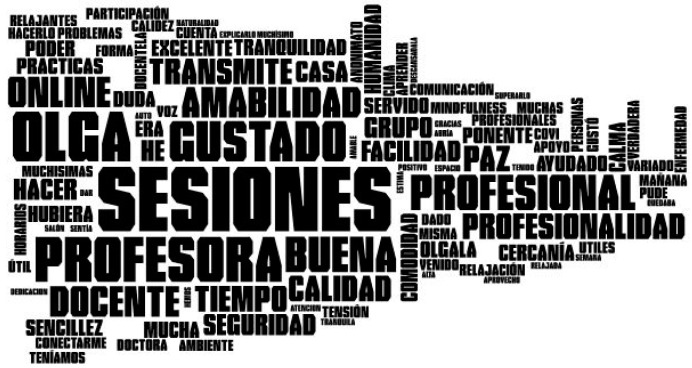
Infographic for the text analyzed corresponding to open question 1 from the data gathering information.

We obtained Figure 11 based on the occurrence frequency of each word. It is clear that the word *sessions* are the one that appears most often in the text analyzed, although the words *teacher* and *good quality* come next in terms of relative frequency. Furthermore, attention should be drawn to the words *situation, professional, professionalism,* and *peace*, among others, in the responses related to question 1.

**Figure 12:**
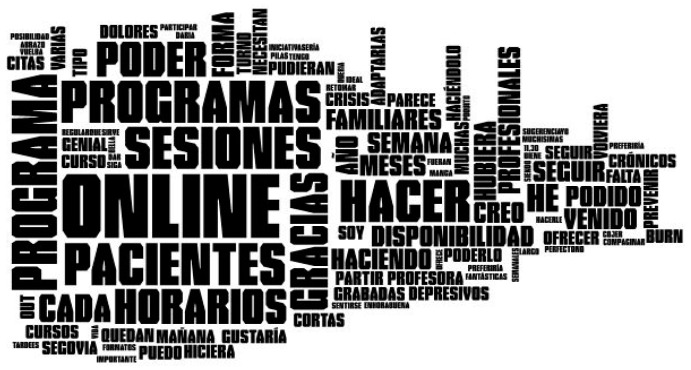
Infographic for the text analyzed corresponding to open question 2.

In the same way, we obtained Figure 12 based on the occurrence frequency of each word. It is clear that the word *online* is the one that appears most often in the text analyzed, although the words *patients* and *sessions* come next in terms of relative frequency. Furthermore, attention should be drawn to the words *program, follow, timetables,* and *thank you,* among others, in the responses related to question 2.

## 4. Discussion

In this section, we shall discuss the model and its structure as an NLP tool for automatic text classification in three sentiment categories. In our case, the amount of available data is limited for the purpose of training a model from scratch, and so this section will focus on the axes of mind comprehension on the part of users, and how the tool helped us understand the raw texts consigned in the survey.

The authors in [46] present different deep learning architectures to perform the sentiment analysis task with notoriously high performances, for different general purpose databases, such as Tweet and Movie database. However, the amount of data they use to train the models is large enough to fit sophisticated architectures such as the proposed Attention-based Bidirectional CNN-RNN Deep Model (ABCDM). As embedding to perform the encoding task, from the text domain to the numeric domain, they use Glove. This work is very interesting, since these pre-trained architectures could be used for us in future works, to be compared with the performance of our proposed approach. However, it is very important to highlight that SWIVEL is an improvement of Glove, as expressed in [40]. Therefore, to make a fairy comparison, the deep learning models would have to be implemented and trained with SWIVEL model too. Finally, we take into account state-of-the-art work to a baseline, but it is not directly comparable because it is not explicitly described which Internet Movie Database was used in any stage of the analysis carried out by the authors.

If we take other studies into account in which some mindfulness practices intervened, the result indicated that the patients had reduced stress and blood pressure [42], while in other mindfulness practices, it was ascertained that it helped with insomnia [43]. In our case, the results obtained regarding patients’ perception in the sentiment analysis conducted using the MPL1 structure provide us with 93.02 accuracy, which would indicate that most of the responses received to this question were considered positive.

In works such as [25], it is shown that the way of tackling the main deficits in the capacity for data mining of registers such as these means that there may be complex and technically interesting innovations when using NPL. In this sense, 72.05% mean accuracy was obtained in question 2, which is not excessively high, and this would seem to indicate that comments were received that might give rise to strategic changes in order to improve the design of the course in the future.

According to [44], data visualization is a powerful mechanism for representing data, which is why words are more frequently found related to the moment when the course was taken in the word cloud generated for question 1. Attention should be drawn to words such as sessions, good quality, and professionalism, these last-mentioned enabling the conclusion to be reached that the course is successful in terms of user satisfaction. For its part, in the word cloud related to question 2, noteworthy results are online patients and timetables which, combined with the results shown in Table 4 regarding the sentiment analysis, enable us to conclude that the impact of these topics was positive in terms of the course.

The authors in [47] propose a sentiment analysis model to analyze a database of tweets between the peak period or impact of the COVID19 disease, with a total of 530,232 text, collected between 9 April 2020 until 15 April 2020. The proposed model is interesting, since it seeks to find a classification of the texts arranged in the tweets and the authors try to identify the idea of sentiment related to the feelings of the people about the Sars Cov 2 pandemic. This is high-impact work because the world is still facing the pandemic. This work is related to ours, in the sense that we want to identify too, with the application of NLP techniques, peoples’ feelings that were caused by the COVID19 pandemic. However, it differs strongly from our work in the following terms: They do not present a confusion matrix, which makes comparison difficult and also opens the possibility of ambiguity, since the ground truth is not clear. On the other hand, in Table 2 they have different intervals associated to a feeling category, but in a hard way. In contrast to our work, only three categories we assumed, and the thresholds of the intervals were adjusted by cross-validation with respect to the ground truth. Finally, we propose the use of a sigmoidal tangent activation function in the final layer, to obtain an output value between −1 and 1, unlike the authors of [47] who propose a Bayesian model, whose output is a probability, as they themselves express it in the manuscript, but keeping the output between −1 and 1. This could lead to confusion, since negative probabilities are not defined in the mathematical literature.

In [48], the authors propose an exhaustive review of the state of the art, where they describe multiple state-of-the-art deep learning models, currently in use for Natural Language Applications, as well multiple datasets for training and testing the proposed architectures. They highlight the use of CNN, LSTM, RNN, and FCNN. This has high relevance for our work, because it ratifies the potential of our approach, in the sense that we use a Fully Connected Neural Network (FCNN) concatenated with a swivel 20 model, for text encoding and classification. In this sense, we agree with the authors who are describing state-of-the-art techniques, such as the use of general purpose IMDB database, which the authors recommend for the time of applications we are carrying out. Finally, we applied the use of this database as a transfer learning model for our task, where the amount of data is small.

## 5. Conclusions

The main conclusion drawn from this study would be that mindfulness techniques have helped to provide health care workers from Castile and Leon with the means to consciously regain control of their thoughts and, although they were subjected to great pressure at work and uncertainty, the course became a tool that helped them retain the concentration required in times of COVID-19.

From the standpoint of natural language processing, it can be concluded that the availability of freely accessible data bases such as IMDB, together with word encoders such as swivel embedding, enable low-cost computer tools to be constructed within a short time, relative to weeks. These can then be used by interdisciplinary researchers who do not have sufficient information at their disposal to construct a text classification model suitable to the context that is being studied. This makes a major contribution, as it opens the doors to the application of deep learning techniques such as transfer learning in different areas of exploratory qualitative research for the automatic analysis of raw text-data.

The experience involved in putting together the online mindfulness course during the first wave of Covid-19 enables us to consider it as having been positive for professionals to gain access to this type of mental health intervention via this sentiment analysis. Participant professionals left us their recommendations about how to continue with this type of intervention and help the instructor to keep up withstanding pressure at work, and also shows us that the importance of this course means it should be continued for such health care professionals.

Below are provided the main limitations found from the standpoint of the technology used to analyze data.

The NLP classification model uses an automatic translator to translate into the English language, as the source text is in Spanish, which might give rise to errors. As a future line of work, a data base could be used to apply transfer learning in Spanish in the word-encoding (embedding) stage and in the text classification stage.

The system’s sensitivity to spelling mistakes in texts written by users makes automatic translation and interpretation of their sentiment difficult on the part of the NLP model.

The model itself contains a huge number of parameters, such as the weights of the SWIVEL vector text encoding model, as well as the weights of the multilayer perceptron classification network, among others. It is very important to note that the study of parameter impact is becoming increasingly important in the field of machine learning, since explainable and interpretable models are required, especially when health care information is being processed. However, the study of the impact of the parameters is proposed as a future work, where we will analyze in detail how the model, once trained, interacts with the input data to give a result in the output. In this case, a value between −1 and 1 that can be interpreted as a sentiment associated with a degree of sentiment strength. This study could be a great contribution to the state of the art in terms of understanding the machine learning and deep learning models.

One of the major limitations of the proposed model is the bias to the IMDB database, since we use transfer learning and pre trained models in all stages, losing the context of the application. In other words, a greater amount of information is required to train a proper model, in order to improve performance and maintain the information of the context of the analyzed responses. So, when a larger amount of data is available, more sophisticated and deeper models could be trained or adjusted for specific sentiment analysis application. We realized in this work, the amount of text available is very important. This limitation is caused by the data collection experiments, which were not originally designed to address natural language processing techniques. Therefore, this is another opportunity for improvement of the work, because we strongly recommend the need to design the data collection instrument, thinking of the type of methodologies to be used to process the collected data.

As future lines of work for this study, new questionnaires are being put together in which questions are adapted to the results obtained in this paper. The number of individuals who will take part in the new questionnaire is expected to be substantially greater, in order to gather sufficient information with a view to training models appropriate for the application being studied—in this case, mindfulness.

## Figures and Tables

**Table 1:** Hyperparameters of the proposed model.

Hyperparameters	Values
Number of Hidden Layers	1–2
Activation Function of Hidden Layers	RELU
Number of Neurons of the Hidden Layers	1–20

**Table 2:** Performance metrics obtained using the classification models proposed.

Model	Dataset	Positive	Mean Positive—Neutral Threshold	Mean Neutral—Negative Threshold	Negative	Mean Accuracy
MLP1	Question 1	1	0	−0.5	−1	93.02%
MLP1	Question 2	1	0	−0.1	−1	72.05%
MLP2	Question 1	1	0	−0.4	−1	90.53%
MLP2	Question 2	1	0	−0.22	−1	70.25%

**Table 3:** Confusion matrix obtained for the set of responses to question 1 using the MLP1 model proposed.

**Predicted**		**Real**					
	**Class**	**Positive**	**Neutral**	**Negative**	**Precision**	**Recall**	**F1 Score**
	**Positive**	78	2	0	0.98	0.95	0.96
	**Neutral**	3	1	0	0.25	0.33	0.29
	**Negative**	1	0	1	0.50	1.00	0.67

**Table 4:** Confusion matrix obtained for the set of responses to question 2 using the MLP1 model proposed.

**Predicted**		**Real**					
	**Class**	**Positive**	**Neutral**	**Negative**	**Precision**	**Recall**	**F1 Score**
	**Positive**	46	13	5	0.72	0.98	0.83
	**Neutral**	0	0	0	NA	NA	NA
	**Negative**	1	0	3	0.75	0.38	0.50

**Table 5:** Confusion matrix obtained for the set of responses to question 1 using the MLP2 model proposed.

**Predicted**		**Real**					
	**Class**	**Positive**	**Neutral**	**Negative**	**Precision**	**Recall**	**F1 Score**
	**Positive**	70	2	0	0.97	0.85	0.91
	**Neutral**	3	1	0	0.25	0.33	0.29
	**Negative**	9	0	1	0.10	1.00	0.18

**Table 6:** Confusion matrix obtained for the set of responses to question 2 using the MLP2 model proposed.

**Predicted**		**Real**					
	**Class**	**Positive**	**Neutral**	**Negative**	**Precision**	**Recall**	**F1 Score**
	**Positive**	39	5	5	0.80	0.83	0.81
	**Neutral**	0	0	0	NA	NA	NA
	**Negative**	8	8	3	0.16	0.38	0.22

## Data Availability

3rd Party Data, Restrictions apply to the availability of these data. Data was obtained from University of Valladolid and are available with the permission of University of Valladolid. To get the permission please contact the authors.

## Appendix A

**Table A1:** Register of the *Mindfulness* Course in times of the Covid-19 Pandemic.

ID	Question	Answers
Item 0	E-mail	Valid email
	Name and surname(s)	Text
	Age	Expressed as a number
	Sex	Female Male
	Post	Nurse TCAE (auxiliary nurse) TCAE (psychiatric auxiliary nurse at HURH (Rio Hortega University Hospital, Valladolid)) Social worker for SACYL (Castile and Leon Health Care Service) Pediatrician Dermatologist Doctor Laboratory technician Physiotherapist Psychologist Psychiatrist Other Midwife Hospital-other Hospital–accident and emergency
1	Awareness of the program and type of contact:	By email By direct contact By telephone Via the traditional system (Interconsulta) Social networks
2	Current work situation (regarding COVID-19):	Active Sick leave Self-isolation Unemployed Other: telecommuting, maternal leave, freelance, vacation
3	Reason for the request	Anxiety Insomnia Depression Psycho-emotional support Other
4	Reason for main concern about Covid-19	Work-related stress Stress owing to family members (not ill with Covid-19) Stress owing to family members (ill with Covid-19) Stress owing to Covid-19 infection Other (concern with social consequences (unemployment, financial situation) regarding the pandemic, emotional control, uncertainty, closeness to vulnerable people, family members who have died from Covid-19), hostility among work colleagues, etc.

**Table A2:** Survey based on Client Satisfaction Questionnaire (CSQ-8).

ID	Question	Answers	
Item 0	Professional health care category		
	Age	Expressed as a number	
	Sex	Female Male	
	Place of work		
Item 1	1. How would you rate the quality of the online emotional mindfulness support service received?	Excellent	
		Good	
		Average	
		Poor	
Item 2	2. Did you receive the type of support you required?	Definitely not On few occasions In general, yes Definitely yes	
Item 3	3. To what extent has this program helped you solve your problems?	Almost entirely Mostly Only to some extent Not at all	
Item 4	4. If a friend needed similar help, would you recommend this program to them?	Definitely not I don’t think so I think so Definitely yes	
Item 5	5. How satisfied are you with the amount of help you have received?	Not satisfied at all Indifferent or moderately satisfied Moderately satisfied Very satisfied	
Item 6	6. Have the services you received helped you deal better with your problems?	Yes, they helped me a lot Yes, they helped me to a certain extent No, they didn’t really help me No, they seemed to make things worse	
Item 7	7. Generally speaking, how satisfied are you with the services you have received?	Very satisfied Moderately satisfied Somewhat satisfied Very unsatisfied	
Item 8	8. If you needed help again, would you return to our program?	Definitely not Possibly not Yes, I think so Yes, for sure	
Item 9	9. About the course: - [You think the duration of the course might have been too short] - [The degree of privacy and respect for intimacy was considered and complied with] - [I felt comfortable during therapy] - [You think the duration of the intervention might have been too short] - [The online system was easy to use] - [Owing to your experience as a health care professional, you consider the program to be suitable and a similar program should be offered to those affected by Covid-19 and/or their family members] - [Owing to your experience with the pandemic, and if there were another similar situation, you think a program like this should be promoted]	I completely agree I agreeI don’t agree I disagree I totally disagree	
Item 10	10. Comparing face-to-face with online intervention, if you had to choose and in light of the experience gained, what would be your preferences (Five [5] would mean indifferent to either type of intervention):	1	Online
		10	Face to-face
Item 11	Optional: Q1-Share with us what you most liked about the attention received. Q2-Share your suggestions and recommendations for improvement with us.	Open responses	
